# Empagliflozin Alleviates Carfilzomib-Induced Cardiotoxicity in Mice by Modulating Oxidative Stress, Inflammatory Response, Endoplasmic Reticulum Stress, and Autophagy

**DOI:** 10.3390/antiox13060671

**Published:** 2024-05-30

**Authors:** Mina Y. George, Mohamed S. Dabour, Eman Rashad, Beshay N. Zordoky

**Affiliations:** 1 Department of Pharmacology and Toxicology, Faculty of Pharmacy, Ain Shams University, Cairo 11566, Egypt; 2 Department of Experimental and Clinical Pharmacology, College of Pharmacy, University of Minnesota, Minneapolis, MN 55455, USA; dabou003@umn.edu (M.S.D.); zordo001@umn.edu (B.N.Z.); 3 Department of Clinical Pharmacy, Faculty of Pharmacy, Tanta University, Tanta 31111, Egypt; 4 Department of Cytology and Histology, Faculty of Veterinary Medicine, Cairo University, Giza 12211, Egypt; emanrashad@cu.edu.eg

**Keywords:** autophagy, carfilzomib, empagliflozin, endoplasmic reticulum stress, multiple myeloma, SGLT-2 inhibitor

## Abstract

Carfilzomib is an irreversible proteasome inhibitor used for multiple myeloma patients. However, carfilzomib treatment is associated with cardiovascular complications. Empagliflozin, an Sodium Glucose Co-transporter 2 inhibitor (SGLT-2) inhibitor, is an oral antidiabetic drug with proven antioxidant and anti-inflammatory properties. The aim of the present study was to determine the cardioprotective effects of empagliflozin against carfilzomib-induced cardiotoxicity. C57BL/6 mice were randomly divided into four groups: control, empagliflozin, carfilzomib, and carfilzomib + empagliflozin. Empagliflozin prevented carfilzomib-induced cardiotoxicity by ameliorating histological alterations, CK-MB, and troponin-I. Moreover, it inhibited carfilzomib-induced oxidative damage and inflammation via its action on catalase activity, reduced glutathione levels and superoxide dismutase activity, and reduced nuclear factor-κB (p65) and cytokine levels. Mechanistically, empagliflozin abrogated endoplasmic reticulum stress induced by carfilzomib, as evidenced by the effect on the Glucose Regulated Protein-78 (GRP-78)/Activating Transcription Factor 6 (ATF6)/C/EBP homologous protein (CHOP) axis. Intriguingly, carfilzomib significantly induced autophagy, an effect that was further enhanced by empagliflozin, evidenced by increased LC3B and beclin-1 mRNA expression and reduced p62 expression. The effect of empagliflozin on apoptosis was confirmed by reduced expression of active caspase-3. Importantly, empagliflozin did not alter the cytotoxic effect of carfilzomib on human U266B1 multiple myeloma cells. our findings suggest that empagliflozin may provide a new therapeutic strategy to mitigate carfilzomib-induced cardiotoxicity in multiple myeloma patients.

## 1. Introduction

Multiple myeloma ranks as the second most prevalent hematologic cancer in the USA [1]. Owing to recent advances in treatment, both clinical response and survival rates have remarkably improved [2]. Among these treatment options, proteasome inhibitors such as bortezomib, carfilzomib (CFZ), and ixazomib have been employed in combination with dexamethasone for the management of multiple myeloma [3]. However, cardiotoxicity has been extensively reported with the use of proteasome inhibitors, especially with CFZ [4]. CFZ-induced cardiovascular toxicity refers to the direct impact on the heart and vasculature structure or function [5].

CFZ is an irreversible proteasome inhibitor used in cases of relapsed/refractory myeloma. It forms a covalent bond with β5 (chymotryptic-like activity) and β5i immunoproteasome, with equivalent potency [6]. When proteasome-mediated proteolysis is inhibited, this results in the accumulation of polyubiquitinated proteins. These proteins disrupt intracellular protein homeostasis, resulting in cell cycle arrest, apoptosis, and inhibition of cancer metastasis [7]. However, CFZ has been reported to cause heart failure (4.1%), arrhythmia (2.4%), ischemia (1.8%), and cardiac arrest [8]. Although the underlying mechanisms of CFZ-induced cardiotoxicity remains unclear, CFZ-induced cardiotoxicity is associated with increased reactive oxygen species (ROS) levels [9], activated nuclear factor-kappa B (NF-κB), and heightened endoplasmic reticulum (ER) stress [10].

The sodium/glucose cotransporter-2 (SGLT-2) inhibitor, empagliflozin (EMPA), was originally approved for type II diabetes mellitus. Subsequent studies have documented its efficacy in mitigating cardiovascular events among diabetic patients [11]. In addition, EMPA has been found to reduce the risk of hospitalization for heart failure and cardiovascular death in type II diabetic patients in the EMPA-REG OUTCOME trial [12]. Recently, EMPA was also approved for management of heart failure in diabetic and non-diabetic patients [13]. The cardioprotective effects of EMPA were mediated by its antioxidant [14] and anti-inflammatory [15] effects. It is worth mentioning that promising cardioprotective effects of EMPA were reported against chemotherapy-induced cardiotoxicities such as doxorubicin [16] and methotrexate [17]. However, its cardioprotective effect has not been reported previously with CFZ. Therefore, our objective was to elucidate the cardioprotective effects of EMPA against CFZ-induced cardiotoxicity, in addition to investigating the potential underlying mechanisms. Additionally, it was imperative to validate that EMPA does not interfere with the anticancer activity of CFZ.

## 2. Materials and Methods

### 2.1. Drugs and Chemicals

Carfilzomib was purchased from MedChemExpress (Monmouth Junction, NJ, USA). EMPA was purchased from Al-Hikma Pharmaceutical Co. (Giza, Egypt). 3-(4,5-dimethylthiazol-2-yl)-2,5-diphenyl-2H-tetrazolium bromide (MTT) was purchased from Millipore Sigma (St. Louis, MO, USA).

### 2.2. Animals

Male C57BL/6 mice (Nile Co. for Pharmaceutical and Chemical Industries (Cairo, Egypt)) 10–12 weeks of age and weighing 25–30 g were purchased from Nile Co. for Pharmaceutical and Chemical Industries, Cairo, Egypt. The mice arrived one week before experimentation to acclimatize. They were housed in cages (4 mice per cage) with dimensions of 80 cm × 40 cm × 20 cm. Mice were kept in an airconditioned atmosphere (25 °C), with alternatively 12 h light and dark cycles. They were fed with animal chow (46.4% carbohydrates, 23.6% protein, 3% fat, and 27% calcium, phosphorous, and fibers). They were given water ad libitum.

### 2.3. Experimental Design

Thirty-two mice were randomly assigned to four groups (eight mice each) and treated for 2 days as follows: Group 1 received vehicle (1% DMSO in purified water for injection) i.p. and oral 0.5% carboxy methyl cellulose (CMC), once per day each for 2 days. Group 2 received EMPA at a dose of 10 mg/kg by oral gavage for 2 consecutive days, in addition to 1% DMSO in purified water for injection (i.p.). Group 3 was administered CFZ at a dose of 8 mg/kg i.p. for 2 consecutive days, in addition to 0.5% CMC daily for 2 days via oral route. Group 4 received CFZ and EMPA with 2 h period differences, at the same dosage regimen as previously mentioned, for 2 consecutive days. Doses were selected based on previous studies [10,18].

On day 3, mice were anesthetized. Then, blood samples were collected. Afterwards, mice were sacrificed. Heart samples from each group were preserved in 10% buffered formalin and then embedded in paraffin for histological examination. In addition, other heart samples were excised and immediately frozen in liquid nitrogen before being stored at −80 °C. The remaining samples were homogenized at a ratio of 1:10 (*w*:*v*) in potassium phosphate buffer with a pH of 7.5 for subsequent biochemical analysis.

### 2.4. Cardiotoxicity Markers

Blood samples were centrifuged, and serum was separated for determination of creatine CM-MB and troponin-I. A CK-MB kit was purchased from Spectrum Co., Cairo, Egypt (Catalogue no.# 239002). Results were expressed as U/L. Troponin I was determined using ELISA kit purchased from Life diagnostics, Inc., West Chester, PA, USA (Catalogue no. CTNI-1-HSP). Results were expressed relative to standard as ng/mL.

### 2.5. Histological Examination

Heart tissues from different groups were fixed in 10% neutral buffered formalin for 24 h. Then, samples were washed with water and dehydrated using serial dilutions of methyl, ethyl, and absolute ethyl alcohols. Afterwards, samples were cleared using xylene and then paraffinized (56 °C—24 h). Heart tissue blocks were cut (4–6 µm thicknesses) using a slide microtome and assembled on glass slides. Hematoxylin and eosin staining was performed. Then, visualization was conducted using a Leica microscope (CH9435 Hee56rbrugg) (Leica Microsystems, Heerbrugg, Switzerland) [19]. 

### 2.6. Determination of Catalase (CAT), Reduced Glutathione (GSH), and SOD Activity

Oxidative stress markers were assessed using kits purchased from Biodiagnostics, Giza, Egypt. Antioxidant catalase (CAT) enzyme cardiac activity was estimated colorimetrically according to the method of Aebi [20]. Enzyme activities were expressed as unit/mg protein. Assessment of reduced glutathione (GSH) was determined colorimetrically in accordance with Beutler et al. [21]. The results were expressed as mmol GSH/mg protein. In addition, SOD activity was measured in accordance with Nishikimi et al. [22]. Results were expressed as U/min/mg protein.

### 2.7. Inflammatory Cytokine Determination

Following nuclear extraction, phosphorylated NF-κB (p65) subunit was assessed using an ELISA kit from Elabscience, Houston, TX, USA (Catalogue No. #E-EL-M0838). Additionally, inflammatory cytokines, including tumor necrosis factor-α (TNF-α) and interleukin-1β (IL-1β), were determined colorimetrically using ELISA kits from Elabscience, USA (Catalogue No. #E-EL-M3063 and E-EL-M0037, respectively) and assessed according to the manufacturer’s protocol. Results were expressed as pg/mg protein.

### 2.8. Determination of ER Stress Markers

ER stress markers—glucose regulated protein 78 (GRP-78), ATF6, and C/EBP homologous protein (CHOP)—were assessed using commercial ELISA kits purchased from Life span biosciences, Seattle, WA, USA (Catalogue No. LS-F7651-1), Novus Biologicals, Centennial, CO, USA (Catalogue No. NBP2-69886), and Sunlong Biotech, Hangzhou, China (Catalogue No. SL0893Mo), respectively, in compliance with the manufacturers’ protocols.

### 2.9. qRT-PCR Determination of Autophagy Markers, LC3B and Beclin-1

qRT-PCR was used to evaluate the mRNA expression levels of LC3B and beclin-1 in cardiac tissues. According to the manufacturer’s protocol, total RNA was extracted from cardiac samples via a QIAamp RNAeasy plus kit, Qiagen, Hilden, Germany. Isolated RNA concentration was assessed using Nanodrop, Ann Arbor, MI, USA (ND-1000 USA). Afterwards, conversion to cDNA was adopted using a ReverAid RT kit, ThermoFisher Scientific, Waltham, MA, USA. Relative gene expression of LC3B and beclin-1 was determined using SYBR^®^ Green PCR Master Mix (Applied Biosystems, Carlsbad, CA, USA) using β-actin as housekeeping gene with Bio-Rad CFX OPUS 96, Tacoma, WA, USA. Denaturation was performed at 95 °C (10 min) and then 40 cycles at 95 °C (15 s). Annealing at 54–58 °C (1 min) was performed. The primer sequences for LC3B, beclin-1, and β-actin were added (Table 1) [23]. Following normalization to the control group and β-actin, mRNA expression levels were determined using the 2^−∆∆Ct^ threshold cycle method [24].

### 2.10. Immunohistochemical Determination of Active Caspase-3 and p62

Immunohistochemistry was performed on paraffin tissue sections and fixed on positively charged slides by using the avidin–biotin–peroxidase complex (ABC) method. Rabbit anti-active caspase-3 monoclonal antibody, clone [EPR18297], unconjugated (Abcam, Cambridge, UK, Cat# ab184787, dilution: 1:1000), and rabbit p62 polyclonal antibody (Elabscience, Cat# E-AB-68411, dil.:1:50) were tested. Sections from each studied group were incubated with the formerly stated antibodies; subsequently, the chemicals involved for the ABC technique (Vectastain ABC-HRP kit, Vector laboratories, Newark, CA, USA) were applied. Marker expression was identified with peroxidase and stained with diaminobenzidine (DAB, produced by Sigma, San Diego, CA, USA) to distinguish the antigen–antibody complex. Negative controls were integrated using non-immune serum instead of the primary or secondary antibodies. Immunostained sections were checked and photographed using a Leica microscope under different magnification powers (CH9435 Hee56rbrugg) (Leica Microsystems, Switzerland). Six high power fields (400×) exhibiting positive brown immunostaining were selected for the quantitative scoring of immunohistochemical results in each serial section of the studied groups. Area % was determined for caspase-3- and p62-stained sections via a Leica QWin 500 image analyzer computer system (London, UK). This image analyzer involved a Leica microscope, a colored video camera, a colored monitor, and the hard disc of a Leica IBM personal computer linked to the microscope and managed by Leica QWin 500 software. Records of each antibody were statistically defined in terms of mean and standard deviation (mean ± S.D.) for the area %.

### 2.11. Cytotoxicity Assay

#### 2.11.1. Cell Culture

Human U266B1 myeloma cells were obtained from ATCC and grown in suspension. U266B1 cells were cultured in ATCC-formulated RPMI-1640 medium (ATCC 30-2001) supplemented with FBS at concentrations of 15% *v*/*v* and 10% *v*/*v*, respectively, along with penicillin (100 U/mL) and streptomycin (100 µg/mL). The cell density was maintained between 1 × 10^5^ and 1 × 10^6^ cells/mL in untreated 75 cm^2^ flasks, and grown in a 5% CO_2_ humidified incubator at 37 °C.

#### 2.11.2. Cell Viability Assay

The MTT assay was utilized to assess cell viability, measuring cellular metabolic activity as an indicator of viability. U266B1 cells were initially seeded in 96-well plates at a cell density of 5 × 10^5^ cells/mL in a volume of 100 µL/well. Cells were treated with 0.5 µM CFZ in the absence or presence of EMPA (1–50 µM). EMPA was added 2 h before adding 0.5 µM CFZ for 24 h. Post treatment, 25 µL of media containing MTT was added to each well to reach a final concentration of 0.5 mg/mL, followed by incubation for 4 h at 37 °C. To solubilize formazan crystals, acidified isopropanol was added, along with shaking for one hour, after which absorbance values were measured at a wavelength of 550 nm using a Biotek microplate reader (Agilent, Santa Clara, CA, USA). Finally, background readings from wells that only contained media were subtracted and the percentage viability relative to control wells considered as having full viability was determined for each treatment group.

### 2.12. Protein Determination

The protein concentration of different samples was determined using the Bradford protein assay kit (Catalogue No. SK3041, Markham, ON, Canada). In summary, tetradentate copper–protein complexes were formed under alkaline conditions. These complexes then reduced the Folin–Ciocalteu reagent, resulting in a water-soluble blue-colored product that was measured at 750 nm. Results are expressed as mg/mL. 

### 2.13. Statistical Analysis

Data are presented as mean ± S.D. Multiple comparisons were performed using one-way ANOVA followed by Tukey’s post hoc test (unless otherwise specified). A 0.05 level of probability was used as the criterion for significance. Statistical analyses and graph sketching were performed using GraphPad Prism software version 9 (GraphPad Software, Inc., La Jolla, CA, USA). 

## 3. Results

### 3.1. Effect of EMPA and CFZ on Cardiotoxicity Markers

**Figure 1:**
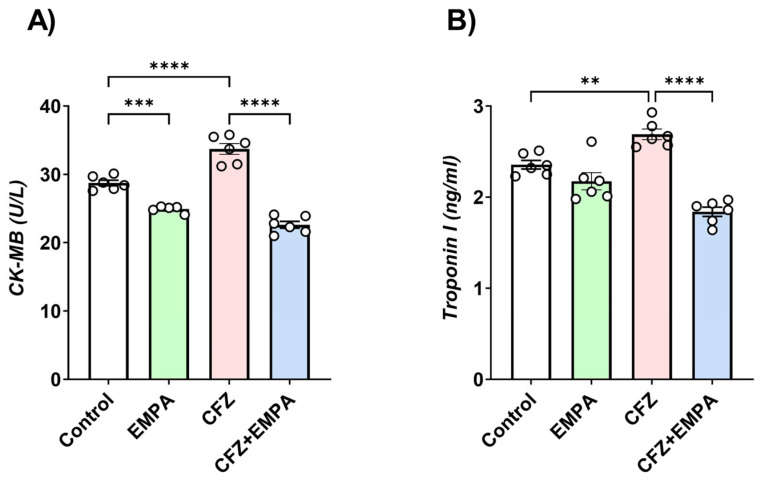
**Effect of EMPA and CFZ on cardiotoxicity markers**. Effect of EMPA and CFZ on serum cardiotoxicity markers: (**A**) CK-MB and (**B**) troponin I determined colorimetrically. Data are presented as mean ± S.D. (*n* = 5–6). **, ***, and ****; statistically significant at *p* < 0.01, *p* < 0.001, and *p* < 0.0001, respectively, using one-way ANOVA followed by Tukey’s post hoc test.

CK-MB and troponin I were assessed to determine the effect of CFZ and EMPA treatment on cardiac functioning. As shown in Figure 1A, CFZ caused a 1.17-fold significant increase in CK-MB concentration relative to the control group. EMPA treatment reduced serum CK-MB levels by 1.49-fold compared with the CFZ-treated group. Furthermore, the EMPA-alone treated group caused a 1.15-fold significant reduction in CK-MB levels relative to the control group. 

In addition, a 1.14-fold significant increase in troponin I levels was detected in CFZ-treated mice relative to the control group. However, CFZ + EMPA-treated mice demonstrated a 1.46-fold significant reduction in troponin I serum levels relative to CFZ-treated mice (Figure 1B).

### 3.2. Histological Examination

**Figure 2:**
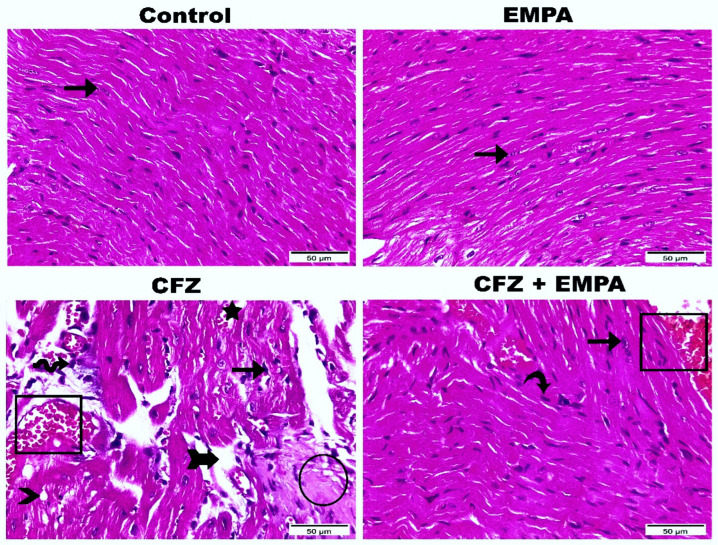
**Effect of EMPA on CFZ-induced histological alterations of cardiac tissues.** Photomicrographs presented histopathological alterations along the heart tissue sections among the studied groups (hematoxylin and eosin stain, magnification power = 400×, and scale bar = 50 µm). Sections from control mice and EMPA-alone-treated mice demonstrated standard myocardial fibers with intact myocytes (arrows). CFZ-treated mice highlighted degenerated and necrotic cardiomyocytes (circle), nuclear pyknosis (arrow), increase in intermuscular spaces (arrow with tail), hemorrhagic spots (star), infiltration of inflammatory cells (wave arrow), fat cells (arrowhead), and congested vasculatures (rectangle). EMPA + CFZ-treated mice disclosed many apparent intact cardiomyocytes (arrow), scarce apoptotic cardiomyocytes (curvy arrow), and congested vasculatures (rectangle).

As illustrated in Figure 2, the control group showed apparent intact histological structures of cardiac wall layers with almost intact well-organized branched cardiomyocytes and intact subcellular details. Also, intact vasculatures without abnormal cellular infiltrates were detected. EMPA-alone-treated mice presented nearly the same features as the control group with intact cardiac myocytes. CFZ-treated mice showed focal areas of degenerated and necrotic cardiomyocytes with nuclear pyknosis, moderate increase in intermuscular spaces with occasional hemorrhagic spots, infiltration of inflammatory cells and fat cells, and congested vasculatures. CFZ + EMPA-treated mice exhibited well-organized histological features of cardiac wall with many apparent intact cardiomyocytes, scarce apoptotic cardiac myocytes, and congested vasculatures.

### 3.3. EMPA Mitigates CFZ-Induced Oxidative Stress in Mice

**Figure 3:**
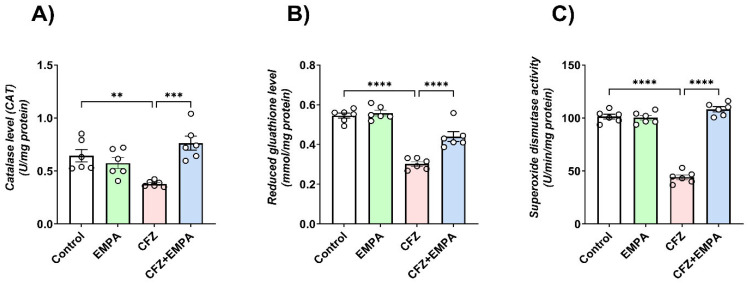
**EMPA mitigated CFZ-induced oxidative stress**. Effect of EMPA and CFZ on cardiac oxidative stress markers: (**A**) CAT, (**B**) GSH, and (**C**) SOD determined using colorimetric methods in mice. Data are presented as means ± S.D (*n* = 6). **, ***, and ****; statistically significant at *p* < 0.01, *p* < 0.001, and *p* < 0.0001, respectively, using one-way ANOVA, followed by Tukey’s post hoc test.

The effect of EMPA and CFZ on oxidative stress markers—catalase (CAT), reduced glutathione (GSH), and superoxide dismutase (SOD)—was assessed in heart tissues, and it was found that CFZ treatment reduced CAT activity in a significant manner relative to the control group by 1.72-fold. By contrast, EMPA treatment significantly enhanced cardiac CAT activity by 1.99-fold compared with the CFZ-treated group (Figure 3A). 

The cardiac level of the antioxidant compound, GSH, was found to be reduced in a significant manner following CFZ treatment compared with the control group by 1.83-fold. On the other hand, EMPA (10 mg/kg) treatment revealed a significant increase in cardiac GSH relative to CFZ-treated mice by 1.5-fold (Figure 3B).

As illustrated in Figure 3C, a 2.33-fold significant decrease in SOD activity was detected in CFZ-treated mice when compared with control mice. However, the group of mice treated with EMPA showed a 2.43-fold significant increase in SOD activity relative to CFZ-treated mice.

### 3.4. Effect of EMPA on the Inflammatory Response Induced by CFZ

The active NF-κB subunit (p65) is considered a cornerstone key in the process of inflammation and triggers the release of inflammatory cytokines. CFZ-treated mice illustrated a 2.43-fold significant increase in p65 NF-κB levels relative to the control group. On the other hand, the EMPA-treated group showed a 1.69-fold significant decrease in p65 NF-κB levels compared with the CFZ-treated group. 

**Figure 4:**
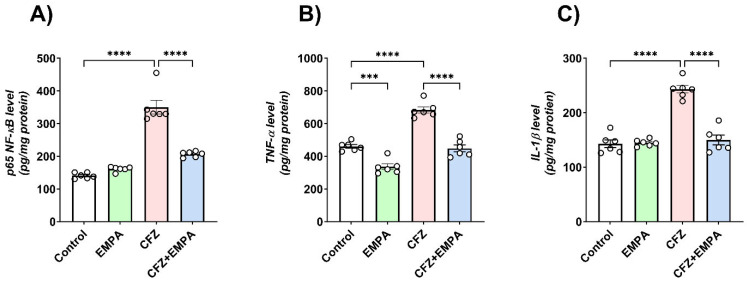
**Effect of EMPA on the inflammatory response induced by CFZ**. Effect of EMPA and CFZ on cardiac inflammatory cytokines: (**A**) p65 NF-κB, (**B**) TNF-α, and (**C**) IL-1β determined by ELISA in mice. Data are presented as mean ± S.D (*n* = 6). *** and ****, statistically significant at *p* < 0.001 and *p* < 0.0001, respectively, using one-way ANOVA, followed by Tukey’s post hoc test.

Furthermore, CFZ treatment enhanced inflammatory cytokine release in cardiac tissue, as evidenced by the significant elevation of TNF-α and IL-1β levels relative to the corresponding control group by 1.48- and 1.7-fold, respectively, while CFZ + EMPA-treated mice exhibited significant reduction in both markers (TNF-α and IL-1β) compared with the CFZ-treated group by 1.52- and 1.62-fold, respectively. Additionally, EMPA-alone-treated mice presented significant reduction in TNF-α levels relative to the control group by 1.37-fold (Figure 4).

### 3.5. ER Stress Signaling Changes Associated with CFZ-Induced Cardiotoxicity

**Figure 5:**
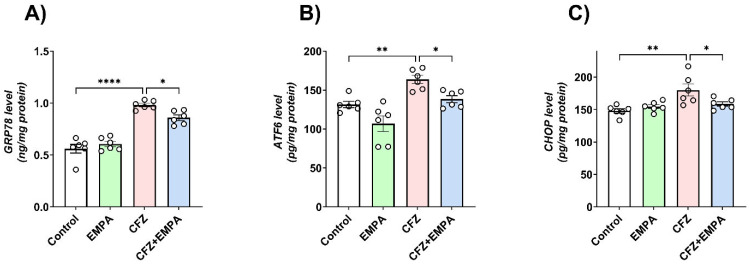
**Effect of EMPA on ER stress induced by CFZ**. Mice were treated with EMPA (10 mg/kg, oral) and CFZ (8 mg/kg) for two consecutive days. Afterwards, blood samples were collected, all mice were sacrificed, and hearts were harvested. Effect of EMPA and CFZ on cardiac levels of (**A**) GRP-78, (**B**) ATF6, and (**C**) CHOP determined by ELISA in mice. Data are presented as mean ± S.D. and analyzed by one-way ANOVA, followed by Tukey’s post hoc test. *, **, and ****, statistically significant at *p* < 0.05, *p* < 0.01, and *p* < 0.0001, respectively.

Levels of GRP-78, ATF6, and CHOP were detected in cardiac tissues to evaluate the effect of EMPA on ER stress (Figure 5). In this context, CFZ-treated mice revealed a significant increase in GRP-78 (Figure 5A), ATF6 (Figure 5B), and CHOP (Figure 5C) relative to the corresponding control group by 1.75-, 1.24-, and 1.22-fold, respectively. On the other hand, the group of mice treated with EMPA illustrated a significant decrease in their cardiac levels compared with CFZ-treated mice by 1.14-, 1.18-, and 1.14-fold, respectively.

### 3.6. Effect of EMPA on Autophagy Markers LC3B, Beclin-1, and p62 Altered by CFZ

**Figure 6:**
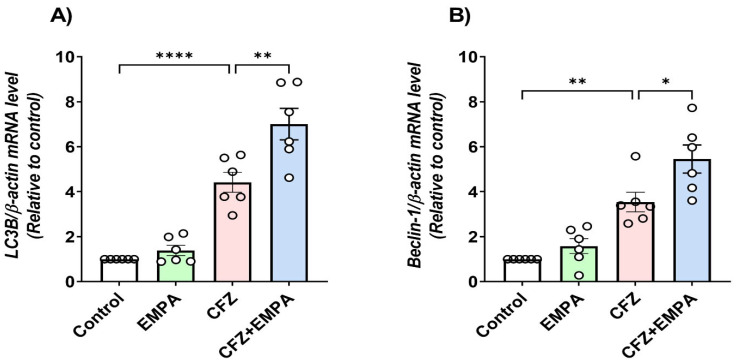
**Effect of EMPA on autophagy markers altered by CFZ**. Mice were treated with EMPA (10 mg/kg, oral) and CFZ (8 mg/kg) for two consecutive days. Afterwards, blood samples were collected, all mice were sacrificed, and hearts were harvested. Thereafter, RNA was extracted and the qRT-PCR gene expression of cardiac; (**A**) LC3B and (**B**) beclin-1 was determined. Data are presented as mean ± S.D (*n* = 6) and analyzed by one-way ANOVA, followed by Tukey’s post hoc test. *, **, and ****, statistically significant at *p* < 0.05, *p* < 0.01, and *p* < 0.0001, respectively.

Autophagy markers LC3B and beclin-1 mRNA expressions were assessed by qRT-PCR. Concerning LC3B (Figure 6A), CFZ-treated mice showed significant elevation in LC3B mRNA expression by 4.42-fold relative to the control group. Treatment with EMPA at a dose of 10 mg/kg further increased LC3B expression, as revealed by a significant increase relative to CFZ-treated mice by 1.59-fold. Regarding beclin-1 gene expression (Figure 6B), a 3.54-fold significant increase was detected when comparing CFZ-treated mice with control mice. However, EMPA treatment showed significant enhancement in beclin-1 mRNA expression by 1.54-fold relative to CFZ-treated mice. 

**Figure 7:**
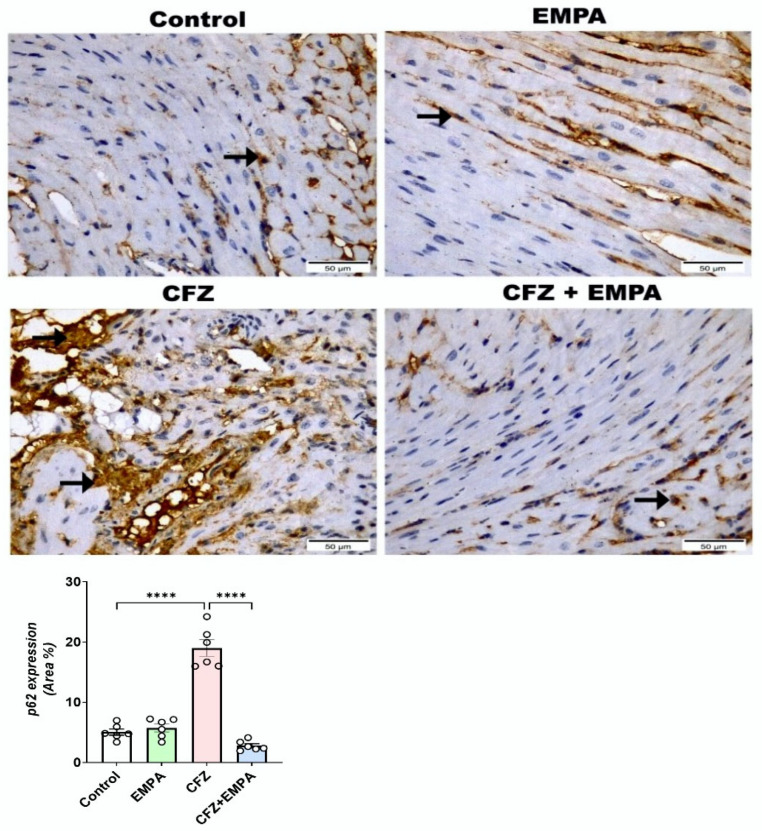
**Effect of EMPA and CFZ on p62 immunohistochemical expression in mice**. Photomicrographs demonstrated the reactivity of p62 in heart tissue sections among the examined groups (magnification power = 400× and scale bar = 50 μm). Heart sections exhibited positive cytoplasmic brown expression as few reactions in the control and EMPA, strong reaction in CFZ, and moderate reaction in CFZ + EMPA. Stained cardiomyocytes were also observed (Black arrow). Data are presented as mean ± S.D. and analyzed by one-way ANOVA, followed by Tukey’s post hoc test. ****, statistically significant at *p* < 0.0001.

Regarding p62 immunohistochemical expression (Figure 7), the heart section from the control group disclosed few positive p62 reactions as cytoplasmic expressions along cardiac myofibers. Almost the same as the control group, the EMPA-alone-treated group also revealed few p62 reactions, without any significance difference between them. The CFZ-treated group highlighted positive p62 cytoplasmic reactivity of cardiac myofibers, with a significant increase compared with the control group. Furthermore, the CFZ + EMPA-treated group displayed moderate p62 positive cytoplasmic reactivity of cardiac myofibers, with a significant decrease compared with the control and CFZ-treated groups.

### 3.7. EMPA Downregulates Caspase-3 Immunohistochemical Induced by CFZ

**Figure 8:**
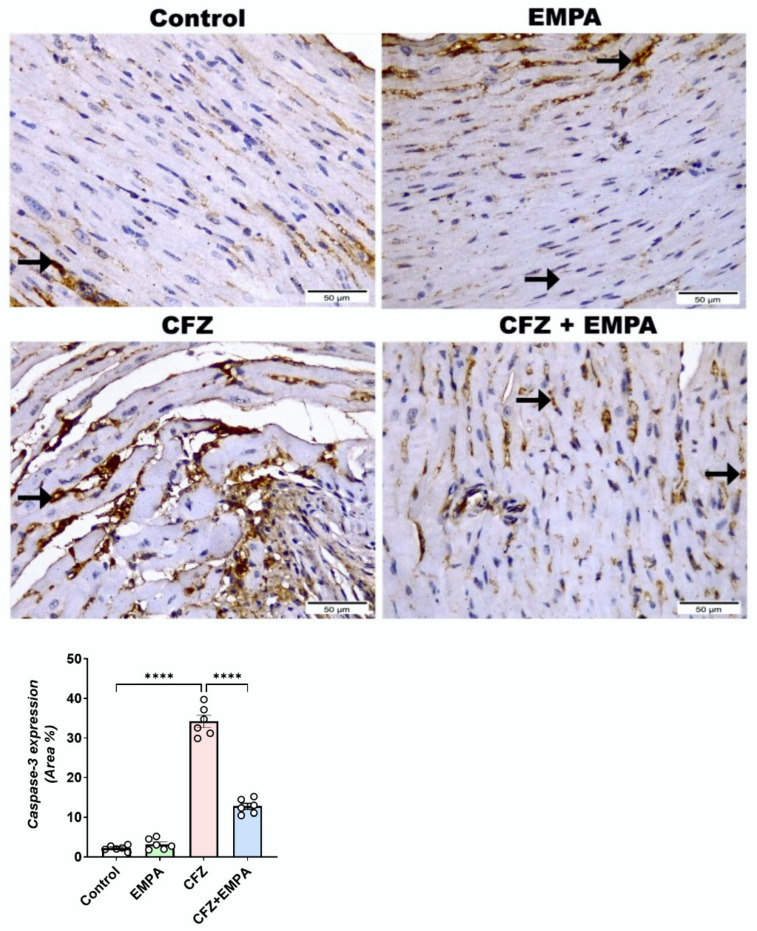
**EMPA downregulated caspase-3 immunohistochemical reaction induced by CFZ**. Photomicrographs displayed the immunohistochemical reactivity of caspase-3 in the heart tissue sections between the inspected groups (magnification power = 400× and scale bar = 50 μm). Heart sections marked positive nuclear brown expression with scarce amount in control and EMPA, high amount in CFZ, and moderate amount in CFZ + EMPA. Stained cardiomyocytes were also observed (Black arrow). Data are presented as mean ± S.D (*n* = 6) and analyzed by one-way ANOVA, followed by Tukey’s post hoc test. ****, statistically significant at *p* < 0.0001.

Since enhanced ER stress, oxidative stress, and inflammation could induce apoptosis, the expression of caspase-3 was assessed in the current study. As illustrated in Figure 8, the heart section from the control group revealed scarce positive caspase-3, appearing as a nuclear expression of cardiomyocytes. Similarly, the EMPA-alone-treated group exhibited scarce expression, like the control group, without any significant difference between them. The CFZ-treated group highlighted the highest positive caspase-3 nuclear reactivity of cardiomyocytes, with a significant difference to the control group. Furthermore, the CFZ + EMPA-treated group displayed moderate positive nuclear reactivity of cardiomyocytes, with a significant decrease compared with the CFZ-treated group. 

### 3.8. EMPA Does Not Interfere with the Anticancer Activity of CFZ

**Figure 9:**
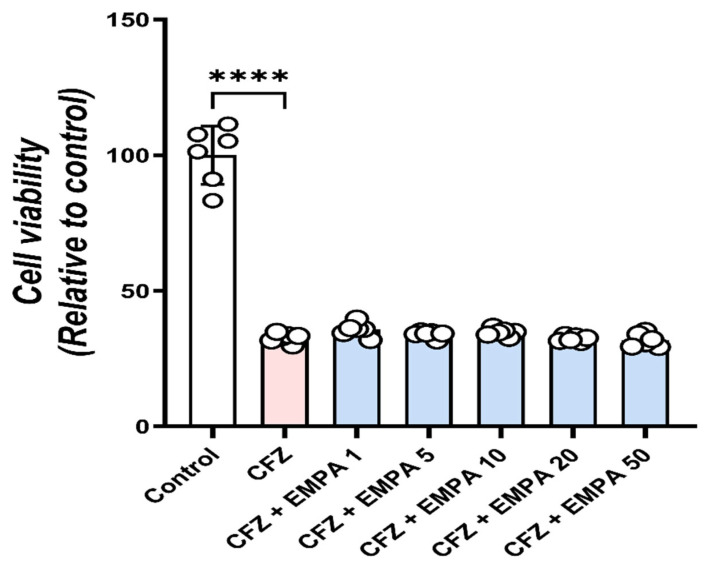
**EMPA did not interfere with the anticancer activity of CFZ.** MTT cytotoxicity assay of CFZ and increasing concentrations (1, 5, 10, 20, 50 µM) of EMPA on human U266B1 myeloma cells. Data are presented as mean ± S.D. (*n* = 6). ****, statistically significant at *p* < 0.0001, respectively, using one-way ANOVA, followed by Tukey’s post hoc test.

Importantly, we assessed whether EMPA impairs the anticarcinogenic efficacy of CFZ in order to contemplate its potential utility as a cardiovascular protective agent during CFZ treatment. U266B1 cells were treated with 0.5 µM CFZ in the absence or presence of EMPA (1–50 µM). The cell viability assay showed that, compared with untreated cells, CFZ significantly reduced the viability of U266B1 cells to nearly 30% (Figure 9). The pre-addition of EMPA did not significantly affect these outcomes, suggesting no interference by EMPA of the anticancer properties exhibited by CFZ.

## 4. Discussion

CFZ, an irreversible proteasome inhibitor, has been utilized in the treatment of multiple myeloma, particularly when combined with dexamethasone [3]. However, several case reports have indicated its possible deleterious effects on heart and vasculatures [25]. The mechanisms behind its cardiotoxicity remain unclear. Studies including 526 patients on CFZ (phase II) concluded that 22% of patients had cardiac adverse effects (13.3% arrhythmia, 7.2% heart failure, 2% cardiomyopathy, and 3% ischemic heart disease) [7]. Furthermore, the cardioprotective effects of SGLT-2 inhibitors against chemotherapy-induced cardiotoxicity have been comprehensively reviewed [26]. The SGLT-2 inhibitor, EMPA, is an oral antidiabetic medication used for type II diabetic patients to control their blood glucose level [27]. It has previously been reported for its cardioprotective activities, regardless of the presence of diabetes [28]. Hence, the aim of the current study was to investigate the mechanisms underlying CFZ-induced cardiotoxicity, assess the cardioprotective effect of EMPA against CFZ-induced toxicity, and explore the potential underlying protective mechanisms.

The 2-day model of CFZ-induced cardiotoxicity was selected based on results from a pilot study study performed on four in vivo models using the same dose of CFZ (8 mg/kg) at different dosing regimens; 2-day model (2 doses of CFZ), 2-day model (2 doses of CFZ) and termination after 2 weeks, 6-day model (4 doses on days 0, 2, 4, and 6), and 2-week model (4 doses on days 0, 1, 7, and 8). The different models were compared in terms of mortality rate, heart index, histological alterations, serum creatine kinase-MB (CK-MB), and troponin I levels (Supplementary Figure S1).

CFZ-induced cardiotoxicity was assessed by estimating serum cardiotoxicity indices and histological examination. Treatment with CFZ led to a significant elevation in serum levels of cardiac enzymes, including CK-MB and troponin I. These cardiotoxicity markers are released in response to cardiac injury and myocardial damage [29]. The effects of CFZ on CK-MB and troponin I were reported previously by Imam et al. [30] and Al-Harbi [31]. This may conclude the acute nature of CFZ-induced cardiotoxicity. On the other hand, EMPA corrected such elevations in CK-MB and troponin I in a significant manner. This may conclude the positive effect of EMPA on cardiac cells [32]. The aforementioned serum biochemical data were further confirmed by histological examination. In our study, CFZ induced histopathological alterations on cardiac tissues, including necrosis with nuclear pyknosis, intermuscular spaces with hemorrhagic areas, inflammatory cell infiltrates, and congested vasculatures. These histopathological changes are in accordance with previous studies [30]. EMPA treatment showed a protective effect on cardiac histological features. In our study, EMPA preserved the histological architecture of heart tissue, further confirming its cardioprotective effect against CFZ-induced cardiotoxicity.

Since oxidative stress and inflammatory response play an important role in CFZ cardiotoxicity [30], we determined the effect of EMPA on both mechanisms. Regarding oxidative stress, CAT, GSH, and SOD levels were detected in heart tissues of mice. CFZ induced a significant reduction in antioxidant enzyme activities of CAT and SOD. In addition, a markedly depleted GSH level was observed following CFZ treatment. This finding may provide insight into oxidative stress as a major underlying mechanism of CFZ-induced cardiotoxic effects. These results are in accordance with Imam et al. [30] and Al-Harbi [31]. In our study, EMPA treatment effectively hindered CFZ-induced oxidative damage, evidenced by its ability to restore CAT and SOD enzyme activities and GSH levels in cardiac tissues of mice. The antioxidant activity of EMPA was reported previously by Wang et al. [33] and Mohammed et al. [34]. Furthermore, Li et al. [14] reported that EMPA may exert its antioxidant properties via ablating NOX4 levels in myocardial tissues of rats via NADPH oxidase activity reduction. Furthermore, they found that the antioxidant activity of EMPA may be attributed to Nrf2/ARE/HO-1 signaling pathway activation. 

The release of ROS triggers an inflammatory response by inducing the phosphorylation, ubiquitination, and subsequent degradation of inhibitory kappa B (IκB), thereby releasing NF-κB, the key mediator of inflammation [35]. Following unmasking, the active subunit (p65) translocates from the cytoplasm to the nucleus, where it promotes the release of inflammatory cytokines, thereby initiating the inflammatory response [36]. Such inflammatory mediators could induce pathological changes in cardiac tissues, precipitating cardiac dysfunction. In the present study, CFZ markedly induced the levels of p65 NF-κB and inflammatory cytokines TNF-α and IL-1β, indicating inflammatory response amplification [9]. This may be attributed to direct NF-κB activation [37]. CFZ was reported previously to induce phosphorylation and degradation of IκB and, hence, activation of NF-κB [38]. However, in the present study, EMPA opposed the inflammatory response induced by CFZ. Previous studies reported the anti-inflammatory activity of EMPA in heart in vivo models [15]. Quagliariello et al. [18] reported that EMPA could improve myocardial strain and reduce cardiac fibrosis and inflammatory cytokines in doxorubicin-treated mice. 

Since there is a crosstalk reported between oxidative stress and ER stress [39], we assessed the ER stress markers GRP-78, ATF6, and CHOP. ER is considered a key player in the process of proteostasis for its role in the folding of damaged proteins. As an irreversible proteasome inhibitor, CFZ has the potential to induce ER stress [10]. ER has been recognized mainly for its role in protein folding and distribution to their final points [40]. In the case of pathological conditions as elevated oxidative stress, ER enters an abnormal state of dysfunction known as ER stress [41]. ER stress could be triggered via three main pathways: PKR-like ER kinase (PERK), activating transcription factor 6 (ATF-6), and inositol requiring enzyme 1α (IRE-1α) [42]. Normally, ATF6 is found to be in an inactivated form via masking with GRP-78. In case of protein misfolding during ER stress, GRP-78 becomes attached to the misfolded proteins, unmasking ATF6. The latter is then released to the Golgi apparatus and dissociated, exposing its functional fragment. This controls many target genes involved in the process of protein folding. Activation of ATF6 stimulates the activation of CHOP, leading to apoptosis of misfolded proteins [43].

CFZ was reported previously to induce ER stress, leading to proteotoxic crisis and cardiotoxicity [10]. Therefore, hindering CFZ-induced ER stress may be a promising solution to restore normal heart functioning and protein control. In the current study, CFZ treatment increased the levels of cardiac ATF6, GRP-78, and CHOP, suggesting the role of ER stress in CFZ-induced cardiac toxicity. Intriguingly, EMPA abrogated CFZ-induced ER stress as indicated by reducing the levels of the aforementioned markers. EMPA was reported recently to hinder ER stress in non-alcoholic fatty liver disease of high-fat diet mice [44]. 

Additionally, autophagy plays a crucial role in maintaining cellular homeostasis, which is stimulated under stressful conditions [45]. Self-eating, known as autophagy, is a cellular dynamic process of protein engulfment and degradation. This leads to discarding misfolded proteins and injured organelles to ensure normal cellular functioning during stressful conditions [45]. The autophagy process begins with autophagosome formation (the structure that fuses with lysosome, forming autophagolysosome to engulf unfolded and misfolded proteins) [46]. Firstly, ATG-1 activates beclin-1 via phosphorylation, which becomes included in a complex needed for autophagosome formation. Secondly, autophagosome elongation occurs upon subsequent activation of other ATG proteins. Afterwards, microtubule-associated protein 1 light chain 3 (LC3) promotes autophagosome elongation. LC3 attachment to the autophagosome stimulates the recruitment of sequestosome-1 or p62 protein. The latter is responsible for transferring abnormal proteins to be degraded in their end destination [47]. 

In our study, CFZ treatment was associated with a marked increase in mRNA expression levels of autophagosome markers LC3B and beclin-1 and an enhanced expression of p62. This may provide evidence of the relation between cardiotoxicity following CFZ treatment and enhanced autophagy. There was an inversely proportional relation reported between ubiquitin–proteasome inhibition and autophagy. Recently, CFZ was reported to enhance the autophagy process, causing vascular toxicity in mice [48]. Moreover, in a mouse model of aging in mice, CFZ promoted an insignificant increased LC3B expression in a 2-day mouse model, while a significant increase in LC3B expression was found in the 4-day model [49]. However, Efentakis et al. [10] reported that CFZ may induce reduction in LC3B expression in a 2-day mouse model of CFZ-induced cardiotoxicity. Furthermore, EMPA treatment was reported previously to induce the autophagy process in a cardiac ischemia/reperfusion animal model via activation of the AMPK-α1/ULK1/FUNDC1 pathway [50]. Our results confirmed these findings. EMPA treatment at a dose of 10 mg/kg significantly enhanced autophagic markers important for autophagosome biogenesis, nucleation, and elongation. Recently, Nasiri-Ansari et al. [44] reported that EMPA treatment could alleviate NAFLD in a mouse model via augmenting autophagy. This may indicate that CFZ treatment induces autophagy as a feedback mechanism for cardiac protection. Interestingly, EMPA treatment enhanced cardiac protection, promoting autophagy, which could be a key element in EMPA-induced cardiac protection. 

Since apoptosis is the destination of all the previously proposed mechanisms, caspase-3 expression was determined following CFZ and EMPA treatment. CFZ was reported previously to induce endothelial apoptosis, as evident by increased caspase-3 expression [51]. On the other hand, EMPA was found to inhibit apoptosis in doxorubicin-induced cardiac toxicity via ameliorating caspase-3 activity [18]. Moreover, Li et al. [52] reported the protective effect of EMPA against diabetic cardiomyopathy through decreasing active caspase-3 protein expression. These were in agreement with our results. CFZ increased caspase-3 expression in a significant manner and was inhibited following EMPA administration. 

Before utilizing EMPA for cardiovascular protection during MM therapy, it is crucial to verify that it does not compromise the antitumor effectiveness of CFZ. Therefore, human MM cells (U266B1) were treated with CFZ alone or in combination with increasing EMPA doses. EMPA does not compromise CFZ ability to inhibit tumor growth, as evident by cell viability assay. This result supports the notion that EMPA can be safely co-administered with CFZ without impeding its therapeutic efficacy against multiple myeloma. Notably, prior research has demonstrated the ability of EMPA to inhibit tumor growth both in vivo and in vitro across various types of cancer [53]. EMPA slowed tumor migration and induced apoptosis of cervical carcinoma [54]. 

## 5. Conclusions

The present study sheds light for the first time on the effect of EMPA on CFZ-induced cardiotoxicity in C57BL/6 mice. Despite its promising effects for multiple myeloma patients, the clinical utility of CFZ is limited by its cardiovascular complications. Importantly, EMPA did not abrogate the anticancer activity of CFZ on human multiple myeloma cells. EMPA showed protective effects against CFZ-induced cardiotoxicity, evidenced by its effect on serum cardiotoxicity markers, histological examination, oxidative stress, and inflammatory cytokines. Mechanistically, treatment with EMPA hindered ER stress and augmented autophagy. Therefore, EMPA may provide a promising clue for maximizing therapeutic benefits of CFZ for multiple myeloma patients worldwide.

## Figures and Tables

**Table 1:** Primer sequences used.

	Forward (Sense)	Reverse (Antisense)	Reference
LC3B	5′-GGGACCCTAACCCCATAGGA-3′	5′-TCTCCCCCTTGTATCGCTCT-3′	[23]
Beclin-1	5′-GCTGTAGCCAGCCTCTGAAA-3′	5′-AATGGCTCCTGTGAGTTCCTG-3′	[23]
β-actin	5′-ACACTCTCCCAGAAGGAGGG-3′	5′-TTTATAGGACGCCACAGCGG-3′	[23]

## Data Availability

The data supporting the conclusions of this article will be made available by the corresponding author on request.

## Supplementary Materials

The following supporting information can be downloaded at: https://www.mdpi.com/article/10.3390/antiox13060671/s1, Figure S1: Pilot study results for different in vivo models of CFZ-induced cardiotoxicity. CFZ dose was 8 mg/kg, i.p. Model (I) represents the 2-day model (2 doses of CFZ on 2 consecutive days). Model (II) represents 14-day model (2 doses of CFZ on 2 consecutive days and termination on day 14). Model (III) represents 6-day model (4 doses of CFZ on days 0, 2, 4, and 6). Model (IV) represents 14-day model (4 doses of CFZ on days 0, 1, 7, and 8). Table (A) shows mortality rate, heart index, and histological alterations following CFZ treatment in different in vivo models. Concerning histological alterations, (--) indicates no damage, (+) indicates mild damage, and (++) indicates moderate damage. Figures (B) and (C) represent serum levels of CK-MB and troponin I, respectively, indicating levels of cardiac damage. Data are presented as means ± S.D. *; statistically significant from control group, respectively, at *p* < 0.05, using one-way ANOVA followed by Tukey–Kramer’s post hoc test.

## References

[1] Kazandjian D. (2016). Multiple myeloma epidemiology and survival: A unique malignancy. Semin. Oncol..

[2] Peterson C., Denlinger N., Yang Y. (2022). Recent Advances and Challenges in Cancer Immunotherapy. Cancers.

[3] Ito S. (2020). Proteasome Inhibitors for the Treatment of Multiple Myeloma. Cancers.

[4] Pokorna Z., Jirkovsky E., Hlavackova M., Jansova H., Jirkovska A., Lencova-Popelova O., Brazdova P., Kubes J., Sotakova-Kasparova D., Mazurova Y. (2019). In vitro and in vivo investigation of cardiotoxicity associated with anticancer proteasome inhibitors and their combination with anthracycline. Clin. Sci. Lond. Engl. 1979.

[5] Sheppard R.J., Berger J., Sebag I.A. (2013). Cardiotoxicity of cancer therapeutics: Current issues in screening, prevention, and therapy. Front. Pharmacol..

[6] Berenson J., Hilger J., Yellin O., Dichmann R., Patel-Donnelly D., Boccia R.V., Bessudo A., Stampleman L., Gravenor D., Eshaghian S. (2014). Replacement of bortezomib with carfilzomib for multiple myeloma patients progressing from bortezomib combination therapy. Leukemia.

[7] VVij R., Siegel D.S., Jagannath S., Jakubowiak A.J., Stewart A.K., McDonagh K., Bahlis N., Belch A., Kunkel L.A., Wear S. (2012). An open-label, single-arm, phase 2 study of single-agent carfilzomib in patients with relapsed and/or refractory multiple myeloma who have been previously treated with bortezomib. Br. J. Haematol..

[8] Waxman A.J., Clasen S., Hwang W.T., Garfall A., Vogl D.T., Carver J., O’Quinn R., Cohen A.D., Stadtmauer E.A., Ky B. (2018). Carfilzomib-Associated Cardiovascular Adverse Events: A Systematic Review and Meta-analysis. JAMA Oncol..

[9] Imam F., Al-Harbi N.O., Al-Harbia M.M., Korashy H.M., Ansari M.A., Sayed-Ahmed M.M., Nagi M.N., Iqbal M., Khalid Answer M., Kazmi I. (2017). Rutin Attenuates Carfilzomib-Induced Cardiotoxicity Through Inhibition of NF-κB, Hypertrophic Gene Expression and Oxidative Stress. Cardiovasc. Toxicol..

[10] Efentakis P., Kremastiotis G., Varela A., Nikolaou P.E., Papanagnou E.D., Davos C.H., Tsoumani M., Agrogiannis G., Konstantinidou A., Kastritis E. (2019). Molecular mechanisms of carfilzomib-induced cardiotoxicity in mice and the emerging cardioprotective role of metformin. Blood.

[11] Heerspink H.J., Perkins B.A., Fitchett D.H., Husain M., Cherney D.Z. (2016). Sodium Glucose Cotransporter 2 Inhibitors in the Treatment of Diabetes Mellitus: Cardiovascular and Kidney Effects, Potential Mechanisms, and Clinical Applications. Circulation.

[12] Fitchett D., Inzucchi S.E., Cannon C.P., McGuire D.K., Scirica B.M., Johansen O.E., Sambevski S., Kaspers S., Pfarr E., George J.T. (2019). Empagliflozin Reduced Mortality and Hospitalization for Heart Failure Across the Spectrum of Cardiovascular Risk in the EMPA-REG OUTCOME Trial. Circulation.

[13] Verma S., Mazer C.D., Fitchett D., Inzucchi S.E., Pfarr E., George J.T., Zinman B. (2018). Empagliflozin reduces cardiovascular events, mortality and renal events in participants with type 2 diabetes after coronary artery bypass graft surgery: Subanalysis of the EMPA-REG OUTCOME® randomised trial. Diabetologia.

[14] Li C., Zhang J., Xue M., Li X., Han F., Liu X., Xu L., Lu Y., Cheng Y., Li T. (2019). SGLT2 inhibition with empagliflozin attenuates myocardial oxidative stress and fibrosis in diabetic mice heart. Cardiovasc. Diabetol..

[15] Koyani C.N., Plastira I., Sourij H., Hallström S., Schmidt A., Rainer P.P., Bugger H., Frank S., Malle E., von Lewinski D. (2020). Empagliflozin protects heart from inflammation and energy depletion via AMPK activation. Pharmacol. Res..

[16] Barış V.Ö., Dinçsoy A.B., Gedikli E., Zırh S., Müftüoğlu S., Erdem A. (2021). Empagliflozin Significantly Prevents the Doxorubicin-induced Acute Cardiotoxicity via Non-antioxidant Pathways. Cardiovasc. Toxicol..

[17] Dogan Z., Ergun D.D., Durmus S., Sahin H., Senturk G.E., Gelisgen R., Senyigit A., Uzun H. (2023). Empagliflozin and sacubitril/valsartan reverse methotrexate cardiotoxicity by repressing oxidative stress and hypoxia in heart embryonic H9c2 cardiomyocytes—The role of morphology of mitochondria observed on electron microscopy. Eur. Rev. Med. Pharmacol. Sci..

[18] Quagliariello V., De Laurentiis M., Rea D., Barbieri A., Monti M.G., Carbone A., Paccone A., Altucci L., Conte M., Canale M.L. (2021). The SGLT-2 inhibitor empagliflozin improves myocardial strain, reduces cardiac fibrosis and pro-inflammatory cytokines in non-diabetic mice treated with doxorubicin. Cardiovascular. Diabetol..

[19] Bancroft J.D., Stevens A. (2016). Theory and Practice of Histological Techniques.

[20] Aebi H. (1984). Catalase invitro. Methods Enzymol..

[21] Beutler E., Duron O., Kelly M.B. (1963). Pharmacologic effect of selenium and antioxidants on liver and kidney of cadmium intoxicated rats. Lab. Clin. Med..

[22] Nishikimi M., Appaji N., Yagi K. (1972). The occurrence of superoxide anion in the reaction of reduced phenazine methosulfate and molecular oxygen. Biochem. Biophys. Res. Commun..

[23] Deng M., Zhong X., Gao Z., Jiang W., Peng L., Cao Y., Zhou Z., Huang L. (2021). Dynamic changes in Beclin-1, LC3B and p62 at various time points in mice with temporary middle cerebral artery occlusion and reperfusion (tMCAO). Brain Res. Bull..

[24] George M.Y., El-Derany M.O., Ahmed Y., Zaher M., Ibrahim C., Waleed H., Khaled H., Khaled G., Saleh A., Alshafei H. (2023). Design and evaluation of chrysin-loaded nanoemulsion against lithium/pilocarpine-induced status epilepticus in rats; emphasis on formulation, neuronal excitotoxicity, oxidative stress, microglia polarization, and AMPK/SIRT-1/PGC-1α pathway. Expert Opin. Drug Deliv..

[25] Wu P., Oren O., Gertz M.A., Yang E.H. (2020). Proteasome Inhibitor-Related Cardiotoxicity: Mechanisms, Diagnosis, and Management. Curr. Oncol. Rep..

[26] Dabour M.S., George M.Y., Daniel M.R., Blaes A.H., Zordoky B.N. (2024). The Cardioprotective and Anticancer Effects of SGLT2 Inhibitors: JACC: CardioOncology State-of-the-Art Review. JACC Cardiooncol..

[27] Nikolaou P.E., Mylonas N., Makridakis M., Makrecka-Kuka M., Iliou A., Zerikiotis S., Efentakis P., Kampoukos S., Kostomitsopoulos N., Vilskersts R. (2022). Cardioprotection by selective SGLT-2 inhibitors in a non-diabetic mouse model of myocardial ischemia/reperfusion injury: A class or a drug effect?. Basic. Res. Cardiol..

[28] Zinman B., Wanner C., Lachin J.M., Fitchett D., Bluhmki E., Hantel S., Mattheus M., Devins T., Johansen O.E., Woerle H.J. (2015). EMPA-REG OUTCOME Investigators. Empagliflozin, Cardiovascular Outcomes, and Mortality in Type 2 Diabetes. N. Engl. J. Med..

[29] Repetto A., Bello B.D., Pasotti M., Agozzino M., Viganò M., Klersy C., Tavazzi L., Arbustini E. (2005). Coronary atherosclerosis in endstage idiopathic dilated cardiomyopathy: An innocent bystander?. Eur. Heart J..

[30] Imam F., Al-Harbi N.O., Al-Harbi M.M., Ansari M.A., Almutairi M.M., Alshammari M., Almukhlafi T.S., Ansari M.N., Aljerian K., Ahmad S.F. (2016). Apremilast reversed carfilzomib-induced cardiotoxicity through inhibition of oxidative stress, NF-κB and MAPK signaling in rats. Toxicol. Mech. Methods.

[31] Al-Harbi N.O. (2016). Carfilzomib-induced cardiotoxicity mitigated by dexrazoxane through inhibition of hypertrophic gene expression and oxidative stress in rats. Toxicol. Mech. Methods.

[32] Wang C.C., Li Y., Qian X.Q., Zhao H., Wang D., Zuo G.X., Wang K. (2022). Empagliflozin alleviates myocardial I/R injury and cardiomyocyte apoptosis via inhibiting ER stress-induced autophagy and the PERK/ATF4/Beclin1 pathway. J. Drug Target..

[33] Wang J., Huang X., Liu H., Chen Y., Li P., Liu L., Li J., Ren Y., Huang J., Xiong E. (2022). Empagliflozin Ameliorates Diabetic Cardiomyopathy via Attenuating Oxidative Stress and Improving Mitochondrial Function. Oxidative Med. Cell. Longev..

[34] Mohammed N.N., Tadros M.G., George M.Y. (2024). Empagliflozin repurposing in Parkinson’s disease; modulation of oxidative stress, neuroinflammation, AMPK/SIRT-1/PGC-1α, and wnt/β-catenin pathways. Inflammopharmacology.

[35] Lingappan K. (2018). NF-κB in Oxidative Stress. Curr. Opin. Toxicol..

[36] Liu T., Zhang L., Joo D., Sun S.C. (2017). NF-κB signaling in inflammation. Signal Transduct. Target Ther..

[37] Habib C.N., Ali A.E., Anber N.H., George M.Y. (2023). Lactoferrin ameliorates carfilzomib-induced renal and pulmonary deficits: Insights to the inflammasome NLRP3/NF-κB and PI3K/Akt/GSK-3β/MAPK axes. Life Sci..

[38] Al-Harbi N.O., Imam F., Al-Harbi M.M., Al-Shabanah O.A., Alotaibi M.R., As Sobeai H.M., Afzal M., Kazmi I., Al Rikabi A.C. (2019). Rutin inhibits carfilzomib-induced oxidative stress and inflammation via the NOS-mediated NF-κB signaling pathway. Inflammopharmacology.

[39] Maamoun H., Benameur T., Pintus G., Munusamy S., Agouni A. (2019). Crosstalk Between Oxidative Stress and Endoplasmic Reticulum (ER) Stress in Endothelial Dysfunction and Aberrant Angiogenesis Associated With Diabetes: A Focus on the Protective Roles of Heme Oxygenase (HO)-1. Front. Physiol..

[40] Desouky M.A., George M.Y., Michel H.E., Elsherbiny D.A. (2023). Roflumilast escalates α-synuclein aggregate degradation in rotenone-induced Parkinson’s disease in rats: Modulation of the ubiquitin-proteasome system and endoplasmic reticulum stress. Chem. Biol. Interact..

[41] Hetz C., Chevet E., Oakes S.A. (2015). Erratum: Proteostasis control by the unfolded protein response. Nat. Cell Biol..

[42] Wolfson J.J., May K.L., Thorpe C.M., Jandhyala D.M., Paton J.C., Paton A.W. (2008). Subtilase cytotoxin activates PERK, IRE1 and ATF6 endoplasmic reticulum stress-signalling pathways. Cell Microbiol..

[43] Awad H.H., Desouky M.A., Zidan A., Bassem M., Qasem A., Farouk M., AlDeab H., Fouad M., Hany C., Basem N. (2023). Neuromodulatory effect of vardenafil on aluminium chloride/D-galactose induced Alzheimer’s disease in rats: Emphasis on amyloid-beta, p-tau, PI3K/Akt/p53 pathway, endoplasmic reticulum stress, and cellular senescence. Inflammopharmacology.

[44] Nasiri-Ansari N., Nikolopoulou C., Papoutsi K., Kyrou I., Mantzoros C.S., Kyriakopoulos G., Chatzigeorgiou A., Kalotychou V., Randeva M.S., Chatha K. (2021). Empagliflozin Attenuates Non-Alcoholic Fatty Liver Disease (NAFLD) in High Fat Diet Fed ApoE^(−/−)^ Mice by Activating Autophagy and Reducing ER Stress and Apoptosis. Int. J. Mol. Sci..

[45] Murrow L., Debnath J. (2013). Autophagy as a stress-response and quality-control mechanism: Implications for cell injury and human disease. Annu. Rev. Pathol..

[46] Russo M., Bono E., Ghigo A. (2021). The Interplay Between Autophagy and Senescence in Anthracycline Cardiotoxicity. Curr. Heart Fail. Rep..

[47] Yu L., Chen Y., Tooze S.A. (2018). Autophagy pathway: Cellular and molecular mechanisms. Autophagy.

[48] Efentakis P., Doerschmann H., Witzler C., Siemer S., Nikolaou P.E., Kastritis E., Stauber R., Dimopoulos M.A., Wenzel P., Andreadou I. (2020). Investigating the Vascular Toxicity Outcomes of the Irreversible Proteasome Inhibitor Carfilzomib. Int. J. Mol. Sci..

[49] Efentakis P., Psarakou G., Varela A., Papanagnou E.D., Chatzistefanou M., Nikolaou P.E., Davos C.H., Gavriatopoulou M., Trougakos I.P., Dimopoulos M.A. (2021). Elucidating Carfilzomib’s Induced Cardiotoxicity in an In Vivo Model of Aging: Prophylactic Potential of Metformin. Int. J. Mol. Sci..

[50] Cai C., Guo Z., Chang X., Li Z., Wu F., He J., Cao T., Wang K., Shi N., Zhou H. (2022). Empagliflozin attenuates cardiac microvascular ischemia/reperfusion through activating the AMPKα1/ULK1/FUNDC1/mitophagy pathway. Redox Biol..

[51] Dabour M.S., Abdelgawad I.Y., Grant M.K.O., El-Sawaf E.S., Zordoky B.N. (2023). Canagliflozin mitigates carfilzomib-induced endothelial apoptosis via an AMPK-dependent pathway. Biomed. Pharmacother..

[52] Li N., Zhu Q.X., Li G.Z., Wang T., Zhou H. (2023). Empagliflozin ameliorates diabetic cardiomyopathy probably via activating AMPK/PGC-1α and inhibiting the RhoA/ROCK pathway. World J. Diabetes.

[53] Lau K.T.K., Ng L., Wong J.W.H., Loong H.H.F., Chan W.W.L., Lee C.H., Wong C.K.H. (2021). Repurposing sodium-glucose co-transporter 2 inhibitors (SGLT2i) for cancer treatment—A Review. Rev. Endocr. Metab. Disord..

[54] Xie Z., Wang F., Lin L., Duan S., Liu X., Li X., Li T., Xue M., Cheng Y., Ren H. (2020). An SGLT2 inhibitor modulates SHH expression by activating AMPK to inhibit the migration and induce the apoptosis of cervical carcinoma cells. Cancer Lett..

