# Supplementary material for: Empagliflozin Alleviates Carfilzomib-Induced Cardiotoxicity in Mice by Modulating Oxidative Stress, Inflammatory Response, Endoplasmic Reticulum Stress, and Autophagy
Source: Antioxidants (Basel). 2024 May 30;13(6):671. doi: 10.3390/antiox13060671 (PMC11200801; doi:10.3390/antiox13060671)
Supplement: Supplementary file 1 [file antioxidants-13-00671-s001.zip › antioxidants-3003784-supplementary.pdf]

antioxidants

MDPI

Supplementary Materials

A

Model	Control	I	II	III	IV
Dosing (CFZ, 8 mg/kg)	------	Two doses on two consecutive days	Two doses on two consecutive days and termination on day 14	Four doses on days; 0, 2, 4, and 6	Four doses on days; 0, 1, 7, and 8
Mortality rate (%)	0%	0%	33.33%	50%	50%
Heart weight/Body weight	4.74 ± 0.095	4.91 ± 0.047	5.22 ± 0.102	5.74 ± 0.121	3.98 ± 0.085
Histological alterations	--	++	+	+	--

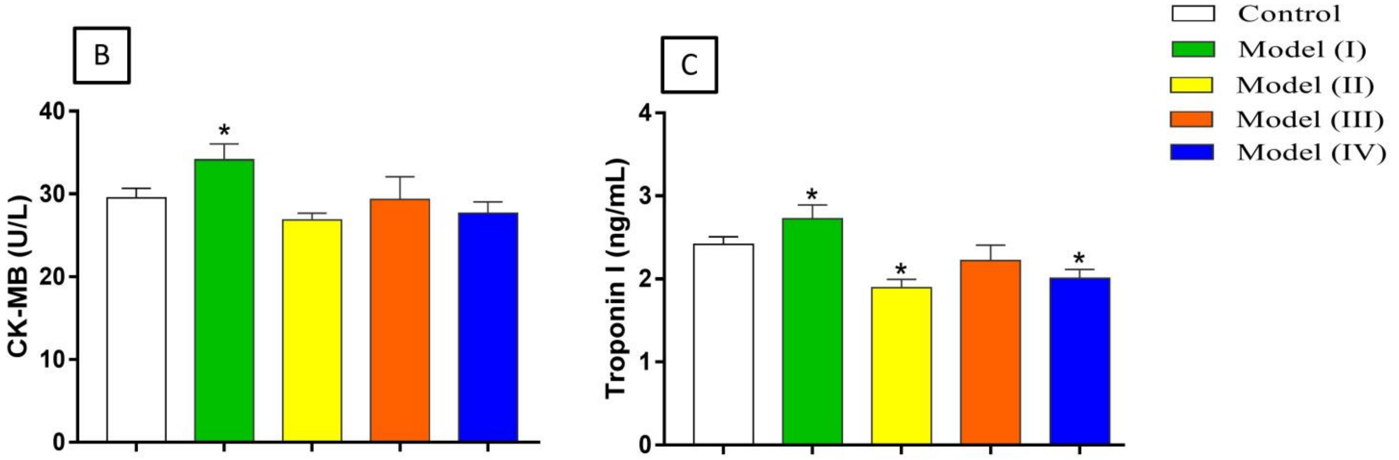

Figure S1. Figure S1: Pilot study results for different in vivo models of CFZ-induced cardiotoxicity. CFZ dose was 8 mg/kg, i.p. Model (I) represents the 2-day model (2 doses of CFZ on 2 consecutive days). Model (II) represents 14-day model (2 doses of CFZ on 2 consecutive days and termination on day 14). Model (III) represents 6-day model (4 doses of CFZ on days 0, 2, 4, and 6). Model (IV) represents 14-day model (4 doses of CFZ on days 0, 1, 7, and 8). Table (A) shows mortality rate, heart index, and histological alterations following CFZ treatment in different in vivo models. Concerning histological alterations, (--) indicates no damage, (+) indicates mild damage, and (++) indicates moderate damage. Figures (B) and (C) represent serum levels of CK-MB and troponin I, respectively, indicating levels of cardiac damage. Data are presented as means ± S.D. *; statistically significant from control group, respectively, at $p < 0.05$, using one-way ANOVA followed by Tukey–Kramer's post hoc test.
